# Sleep problems in autistic children and adolescents: an age-stratified approach

**DOI:** 10.3389/fpsyg.2026.1715093

**Published:** 2026-03-04

**Authors:** Antonio Paone, Maria Grazia Logrieco, Silvia Guerrera, Elisa Fucà, Laura Casula, Alessandra Minutolo, Stefano Vicari, Giovanni Valeri

**Affiliations:** 1 Child and Adolescent Neuropsychiatry Unit, Bambino Gesù Children's Hospital, IRCCS, Rome, Italy; 2 Department of Humanities, University of Foggia, Foggia, Italy; 3 Department of Life Science and Public Health, Università Cattolica del Sacro Cuore, Rome, Italy

**Keywords:** adaptive functioning, age-specific analysis, autism spectrum disorder, behavioral problems, parenting stress, sleep disorders

## Abstract

Sleep disorders are common in autistic children and adolescents. However, the associations between children’s age and clinical features remain underexplored. This study investigates age-specific relationships between sleep difficulties and autism symptomatology, behavioral/emotional problems, cognitive development, and parenting stress. A total of 218 autistic participants were divided into three age groups: 6–36 months, 3–6 years, and 6–18 years. Sleep was assessed using the *Sleep Disturbance Scale for Children* (SDSC); behavioral and emotional difficulties were measured using the *Child Behavior Checklist* (CBCL); autism severity was assessed using the *Autism Diagnostic Observation Schedule – Second Edition* (ADOS2), cognitive development was assessed using the Intelligence Quotient (IQ)/Developmental Quotient (DQ) scores, and parenting stress was assessed using the *Parenting Stress Index* (PSI). Results demonstrate age-specific variations in the relationship between sleep disturbances and clinical features in autistic children. In the 6–36 months group, sleep difficulties were influenced by autism severity, cognitive level, and emotional-behavioral factors. In the 3–6-year-old group, behavioral and emotional regulation issues were associated with sleep problems. In children aged 6–18 years, autism severity, adaptive functioning, and parenting stress emerged as key factors contributing to sleep disturbances. These findings underscore the dynamic and evolving nature of sleep problems in autism, highlighting the need for age-tailored interventions.

## 1 Introduction

Autism Spectrum Disorder (ASD) is a neurodevelopmental condition characterized by impairments in social communication and by repetitive or restricted patterns of behavior or interests (American Psychiatric Association, 2022), with symptoms differing among individuals across a range of severity (Lord et al., 2018; Masi et al., 2017). Core symptoms include difficulties initiating and sustaining social interactions, difficulties understanding social cues, and repetitive or stereotyped behaviors. Non-verbal communication is often impaired, with reduced eye contact, gestures, and facial expressions. Additionally, delays in language development and repetitive speech patterns are commonly observed (Singhi and Malhi, 2023; Cakir et al., 2020).

Autism spectrum disorder is one of the most common neurodevelopmental disorders, with a prevalence that has increased over the last few decades due to improved awareness, early identification, and advances in diagnostic practices (Al-Mamari et al., 2019; Zeidan et al., 2022; Maenner et al., 2023). Epidemiological research shows that approximately 1 in 36 children are diagnosed with ASD, with boys being more frequently affected than girls. Discrepancies in prevalence are influenced by factors such as study methodologies, biological factors, and social determinants, including socioeconomic status (SES), geography, and ethnicity (Zeidan et al., 2022; Maenner et al., 2023).

Children with ASD often experience co-occurring psychiatric and behavioral challenges. Research suggests that approximately 70% of autistic children have at least one comorbid psychiatric disorder, while nearly 40% may have two or more comorbid psychiatric disorder (Bauman, 2010; Barlattani et al., 2023; DeFilippis, 2018; Hossain et al., 2020). Common comorbidities include anxiety, depression, attention-deficit/hyperactivity disorder, and intellectual disability, which can further influence social functioning, adaptive behavior, and overall quality of life (DeFilippis, 2018; Hossain et al., 2020; Al-Beltagi, 2021).

Sleep disorders represent one of the most frequent comorbidities in ASD, affecting approximately 80% of children (Al-Beltagi, 2021; Fatemeh et al., 2022; Burman et al., 2023; Mazzone et al., 2018; Elrod et al., 2016; Aathira et al., 2017; Lai et al., 2019). The most frequent sleep disorder in autistic children is insomnia, which is characterized by difficulty falling asleep, frequent nighttime awakenings, and resistance to going to bed (Berloco et al., 2024). Other sleep disorders, such as parasomnias and excessive daytime sleepiness, have been linked to behavioral challenges and the severity of ASD in several studies (Fadini et al., 2015; Tudor et al., 2012; Bernardi et al., 2023; Mazurek and Sohl, 2016; Goldman et al., 2011; Goldman et al., 2009; Allik et al., 2006).

Sleep cycles undergo significant changes from early childhood through adolescence, reflecting maturation of neural circuits and circadian regulation. In early life, sleep is initially polyphasic, with short cycles and a high proportion of rapid eye movement (REM) sleep, which gradually differentiates into distinct REM and non-REM stages. Over the first years, sleep consolidates into a predominantly nocturnal, monophasic pattern. Across childhood and adolescence, total sleep duration gradually declines, slow-wave sleep (SWS) decreases, and lighter non-rapid eye movement (NREM) stages become more prominent. In contrast, nocturnal sleep becomes increasingly consolidated and structured. These normative developmental patterns are also evident in objective measures, such as age-related decreases in the proportion of REM sleep, increased sleep consolidation, and shifts in electroencephalogram (EEG) spectral power between NREM and REM sleep, consistent with ongoing synaptic pruning and cortical maturation (Carskadon and Dement, 2011; McLaughlin Crabtree and Williams, 2009; Lenehan et al., 2023). During early childhood, REM sleep declines while NREM sleep becomes more differentiated, with slow-wave sleep (SWS) emerging and gradually decreasing over time (Lenehan et al., 2023).

Polysomnographic studies suggest that autistic children frequently exhibit altered sleep architecture, including changes in REM and slow-wave sleep, reduced sleep efficiency, and increased nighttime fragmentation. These sleep patterns have been linked to behavioral profiles and the severity of core ASD symptoms. Meta-analytic evidence indicates that autistic children show differences in sleep stage distribution and efficiency relative to typical development, although these alterations vary by age, symptom severity, and individual clinical features (Chen et al., 2021; Kawai et al., 2023; Nguyen et al., 2022).

Since sleep plays a crucial role in regulating behavior and emotions, disrupted or insufficient sleep may have cascading effects across multiple domains, including the exacerbation of core symptoms of ASD, impairments in adaptive functioning, behavioral regulation, and cognitive processes (Burman et al., 2023; Xavier, 2021). As a matter of fact, a growing body of evidence suggests a bidirectional link between sleep disorders and behavioral/emotional difficulties: in other words, behavioral issues can either intensify or be intensified by sleep disorders, leading to several difficulties, such as anxiety and behavioral and emotional symptoms (Barlattani et al., 2023; Berloco et al., 2024; Taylor et al., 2012). In line with this perspective, Mazurek and Sohl found that specific types of sleep disorders in autistic children, such as night awakenings, were associated with increased levels of physical aggression, irritability, inattention, and hyperactivity (Mazurek and Sohl, 2016), leading to increased levels of parenting stress, as reported in other studies (Berloco et al., 2024; Johnson et al., 2018).

Within this framework, sleep disturbances in ASD are commonly conceptualized as clinical outcomes influenced by multiple child-related characteristics, autistic symptom severity (ADOS), behavioral and emotional difficulties, cognitive functioning, and adaptive skills. Previous studies have consistently reported associations between these domains and increased sleep difficulties across development (Mazurek and Sohl, 2016; Goldman et al., 2011; Sikora et al., 2012; Cohen et al., 2014; Meltzer and Mindell, 2008; Mignolli et al., 2022). Although bidirectional associations have been described, most studies have relied on large, mixed-age samples, often overlooking age-specific patterns, despite evidence that different neurobiological, emotional, and contextual needs characterize distinct developmental stages. Therefore, the present study adopted an age-stratified approach to investigate sleep disturbances across developmental stages in autistic children.

Specifically, the aims were to: (i) examine the prevalence and profile of sleep disturbances across age groups; (ii) explore age-specific associations between sleep disorders and clinical features, including ASD severity, cognitive functioning, adaptive behavior, emotional–behavioral problems, and parenting stress; and (iii) evaluate the relative contribution of neurodevelopmental and psychosocial factors to sleep outcomes within each age group.

## 2 Materials and methods

### 2.1 Participants

A total of 218 children and adolescents were included in the present study. The participants were assessed through neuropsychological and clinical evaluations at the Child and Adolescent Neuropsychiatry Unit of Bambino Gesù Children’s Hospital in Rome between January 2022 and March 2023. Participants were selected from children diagnosed with ASD according to the *Diagnostic and Statistical Manual of Mental Disorders – Fifth Edition, Text Revision* (DSM-5 TR) criteria. Diagnoses were established by a multidisciplinary team comprising child psychiatrists and research psychologists with clinical training. Only those who completed the full assessment process were included in the final sample.

Exclusion criteria included neurological conditions such as epilepsy, encephalitis, brain tumors, and cerebrovascular disorders, as well as language barriers that hindered parental completion of the required questionnaires. The sample included all participants who had not assumed psychoactive or sleep-influencing medications at the time of the evaluation.

For the analysis, the sample was divided into three subgroups based on the version of the *Sleep Disturbance Scale for Children* (SDSC) questionnaire (Bernardi et al., 2023; Bruni et al., 1996; Romeo et al., 2021)—56 infants and toddlers aged 6–36 months (44 boys and 12 girls), 106 children aged 3–6 years (87 boys and 19 girls), and 55 children aged 6–18 years (45 boys and 10 girls). The study’s purpose was thoroughly explained to all caregivers, and informed consent was obtained. This study adhered to the principles outlined in the Declaration of Helsinki and received approval from the local Ethics Committee (protocol code: 2423_OPBG_2021; approved on 27 October 2021).

### 2.2 Measures

A comprehensive, multidisciplinary assessment was conducted using a blend of clinician-administered standardized instruments and parent-reported questionnaires. Cognitive functioning (Leiter−3, *Wechsler Intelligence Scale for Children – Fourth Edition* [WISC-IV], and Griffiths-III) and autistic symptom severity (*Autism Diagnostic Observation Schedule – Second Edition* [ADOS2]) were directly assessed by trained clinicians using standardized instruments. Parent-reported instruments were used to evaluate adaptive behavior (*Adaptive Behavior Assessment System II* [ABAS-II]), behavioral and emotional problems (*Child Behavior Checklist* [CBCL]), sleep disorders (SDSC questionnaires), and parenting stress (Parenting Stress Index [PSI]). All data were collected within a multidisciplinary clinical framework, integrating information from medical history, direct observation, standardized clinical assessments, caregiver interviews, and clinical consultations, with questionnaires used as a complementary source of information. This multimethod approach allowed integration of objective, clinician-observed measures with caregiver-reported information, providing a comprehensive characterization of the child’s developmental, behavioral, and clinical profile.

#### 2.2.1 Cognitive assessment

Cognitive development was assessed using standardized, age-appropriate instruments (Koch et al., 2013). The Leiter-3 provides a non-verbal measure of intelligence obtained through four subtests: Figure/Ground, Form Completion, Classification and Analogies, and Sequential Order, and can be administered from 3 to 75 years of age (Koch et al., 2013). For children younger than 2 years of age or those unable to complete the Leiter-3 due to limited attention span, developmental functioning was assessed using the Griffiths Mental Development Scales – Extended Revised (GMDS-ER) (Griffiths et al., 2006) or the third edition of the Griffiths Scales of Child Development (Griffiths III), both administered from birth to 6 years (Green et al., 2016). The GMDS-ER provides a measure of development in children aged 0–2 years in five different domains (Locomotor, Personal–Social, Language, Eye and Hand Coordination, and Performance). The mean of the six subscale quotients yields the Global Developmental Quotient. The Griffiths III provides a measure of children’s development in five domains: Foundations of Learning, Language and Communication, Eye and Hand Coordination, Personal–Social–Emotional, and Gross Motor. The average of the five quotients yields the subscales, which provide a Global Developmental Quotient (DQ).

The *Wechsler Intelligence Scale for Children – Fourth Edition* (WISC-IV), standardized for children aged 6 to 16 years (Wechsler, 2012), was administered to all participants without language impairments, such as non-verbal or minimally verbal children with significant difficulties in comprehension or production. Minimally verbal children with ASD are typically defined as those who do not develop functional spoken language and remain with limited expressive vocabulary beyond the age of 5 (Guerrera et al., 2025). The instrument comprises 10 core subtests: Block Design, Similarities, Digit Span, Picture Concepts, Coding, Vocabulary, Letter–Number Sequencing, Matrix Reasoning, Comprehension, and Symbol Search. WISC-IV administration provides four different indexes: Verbal Comprehension Index, Perceptual Reasoning Index, Working Memory Index, and Processing Speed Index.

For the analyses, non-verbal IQ (Leiter-3), Full-Scale IQ (WISC-IV), and Developmental Quotient (Griffiths III/GMDS-ER) were used as indices of general cognitive functioning.

#### 2.2.2 Autistic symptoms assessment

The clinical diagnosis of ASD was confirmed using a widely recognized “gold standard” tool, the *Autism Diagnostic Observation Schedule* – *Second Edition* (ADOS2) (Lord et al., 2013).

The ADOS2 is a semi-structured assessment tool designed for a systematic and standardized evaluation of ASD symptoms. The instrument consists of five modules: the Toddler Module, for children aged 12–30 months who do not yet use phrases; Module 1, for children aged 31 months and older who are non-verbal; Module 2, for children with limited verbal communication; Module 3, for children and young adolescents who speak fluently; and Module 4, for older adolescents and adults who also have fluent speech. The assessment was conducted and scored by ADOS examiners who have been trained to maintain research reliability.

ADOS2 calibrated severity scores (ADOS2 CSS) were also analyzed (Gotham et al., 2009). ADOS2 Restricted and Repetitive Behavior Calibrated Severity Score (ADOS2-RRB CSS), ADOS2 Social Affect Calibrated Severity Score (ADOS2-SA CSS), and ADOS2 Calibrated Severity Score based on raw total scores (ADOS2 CSS TOT) were included in the analyses. A strong test–retest reliability of the ADOS-2 CSS across all ADOS Modules was reported (Janvier et al., 2022).

#### 2.2.3 Adaptive behavior assessment

To evaluate adaptive behaviors, the *Adaptive Behavior Assessment System II* (ABAS-II) (Harrison and Oakland, 2003). In the present study, we considered the General Adaptive Composite score, Social Adaptive Composite Score, Conceptual Adaptive Composite score, and Practical Adaptive Composite score of ABAS-II for the analyses. The conceptual domain encompasses abilities related to communication and functional academic performance. The social domain encompasses skills required for engaging in interpersonal interactions, initiating and sustaining relationships, and effectively interpreting social information. The practical domain involves proficiency in activities of daily living and the management of household responsibilities (Harrison and Oakland, 2003).

#### 2.2.4 Behavioral and psychological assessment

To explore the presence of co-occurring behavioral and emotional symptoms, we employed the *Child Behavior Checklist* (CBCL) for children aged 1.5–5 years (Achenbach and Rescorla, 2001) and 6–18 years (Achenbach, 2011). The CBCL is recognized as a reliable instrument for identifying behavioral and emotional difficulties in children and adolescents, and it is frequently used in populations with neurodevelopmental disorders (Althoff et al., 2010; Aitken et al., 2019). In autistic children, the instrument has been suggested for supporting the diagnosis (Pandolfi et al., 2014; Hanratty et al., 2015). Raw scores were transformed into T-scores. All T scores were included in the analysis.

#### 2.2.5 Parenting stress assessment

Parenting stress was assessed using the *Parenting Stress Index*—Short Form (PSI-SF) (Abidin, 1996). This index includes three subscales: Parenting Distress (PD), Parent–Child Dysfunctional Interaction (P-CDI), and Difficult Child (DC). PD subscale measures the level of stress a parent experiences due to personal factors, such as feelings of incompetence or lack of social support. The P-CDI subscale evaluates the parent’s perception of their relationship with their child. The DC subscale assesses the parent’s perception of their child’s temperament and behavior (Abidin, 1996).

The PSI demonstrates strong test–retest reliability (0.96). By summing the item scores, a total PSI score can be calculated. All scores obtained in the main subscales were included in the analysis.

#### 2.2.6 Sleep disorders assessment

To investigate the presence of sleep disorders, the *Sleep Disturbance Scale for Children* (SDSC) was assessed (Bruni et al., 1996; Romeo et al., 2021). The questionnaire is typically completed by caregivers and assesses key areas such as difficulties initiating and maintaining sleep, sleep-disordered breathing, arousal disorders, sleep–wake transition disorders, excessive daytime sleepiness, and excessive sweating during sleep (hyperhidrosis). The SDSC has demonstrated strong psychometric properties, with an internal consistency ranging from 0.71 to 0.79, a test–retest reliability of 0.71, and a high diagnostic accuracy of 0.91 (Bruni et al., 1996).

The SDSC questionnaire was administered according to participants’ age.

SDSC 6–36 months (Romeo et al., 2021) includes 19 items and the following subscales: Disorders of Initiating Sleep (DIS); Disorders of Maintaining Sleep (DMS); Sleep Breathing Disorders (SBD); Parasomnias (PARA); Disorders of Arousal and Sleep Wake Transition (DSWT); Disorders of Excessive Somnolence (DOES); Sleep Hyperhidrosis (SHY).

SDSC 3–6 years (Romeo et al., 2013) includes 26 items and the following 6 subscales: Disorders of Initiating and Maintaining Sleep (DIMS); Sleep Breathing Disorders (SBD); Parasomnias (PARA); Disorders of Excessive Somnolence (DOES); Sleep Hyperhidrosis (SHY); Nonrestorative Sleep (NRS).

SDSC 6–18 years (Bruni et al., 1996) includes 26 items and 6 subscales representing the most common areas of sleep disorders in childhood and adolescence: Disorders of Initiating and Maintaining Sleep (DIMS); Sleep Breathing Disorders (SBD); Disorders of Arousal (DA) such as sleepwalking, sleep terrors, nightmares; Sleep–Wake Transition Disorders (SWTD) such as hypnic jerks, rhythmic movement disorders, hypnagogic hallucinations, nocturnal hyperkinesia, bruxism; Disorders of Excessive Somnolence (DOES); Sleep Hyperhidrosis (SHY).

The SDSC provides a T-score for each subscale and a total score (SDSC Total Score). A *T*-score of 60 or higher indicates a high or clinically significant score, indicating the presence of a sleep disorder.

### 2.3 Plan of analysis

All data were uploaded to SPSS version 27 for analysis (IBM Corp, 2020).

A multivariate model, including bootstrap resampling (1,000 samples; 95% confidence interval), was computed to evaluate the associations between clinical features and sleep variables across age groups as assessed by the SDSC questionnaire. Sleep variables derived from the SDSC were treated as dependent variables. At the same time, autism severity, cognitive functioning, adaptive behavior, behavioral/emotional problems, and parenting stress were entered as predictors, in line with a developmental framework conceptualizing sleep disturbances as clinical outcomes associated with neurodevelopmental and psychosocial features. Given the cross-sectional design, analyses were intended to examine theoretically driven associations rather than causal relationships.

In particular, the model for the 6–36-month age group evaluated the effect of adaptive functioning, autistic symptoms, cognitive functioning, and behavioral problems on the variables DIS, DMS, SBD, PARA, DOES, SHY, and TOT. The model related to the 3–6-year age group evaluated the effects of adaptive functioning, autistic symptoms, cognitive functioning, and behavioral problems on the variables PARA, DIMS, SBD, DOES, SHY, NRS, and TOT. The model for the 6–15-year age group evaluated the effects of the adaptive functioning, autistic symptoms, cognitive functioning, and behavioral problems on the DIMS, SBD, DA, DSWT, DOES, IPN, and TOT.

## 3 Results

### 3.1 Descriptive statistics

Descriptive statistics were used to summarize children’s performance across developmental, behavioral, and adaptive functioning domains. The data were systematically organized by age group: 6–36 months (Table 1), 3–6 years (Table 2), and 6–18 years (Table 3).

**Table 1:** The 6–36 months descriptive statistics.

Variable	Mean	Standard error
SDSC-TOT	51.09	1.17
ADOS2-TOT	15.49	0.34
CBCL-TOT problems	57.86	0.91
ABAS-GAC composite	61.63	1.25
ABAS-DAC composite	67.86	2.70
ABAS-DAS composite	67.18	1.11
ABAS-DAP composite	66.17	1.22
IQ/DQ	65.19	1.80

**Table 2:** The 3–6 years descriptive statistics.

Variable	Mean	Standard error
SDSC-TOT	51.54	1.12
ADOS2-TOT	15.49	0.34
CBCL-TOT Problems	57.86	0.91
ABAS-GAC composite	61.48	1.23
ABAS-DAC composite	67.69	2.65
ABAS-DAS composite	66.94	1.10
ABAS-DAP composite	66.05	1.21
IQ/DQ	65.19	1.80

**Table 3:** The 6–18 descriptive statistics.

Variable	Mean	Standard error
SDSC-TOT	53.95	1.55
ADOS2-TOT	15.49	0.34
Md_PSI_STRESS T	81.79	1.68
CBCL-TOT problems	60.63	1.09
ABAS-GAC composite	61.52	1.23
ABAS-DAC composite	67.65	2.63
ABAS-DAS composite	66.88	1.10
ABAS-DAP composite	66.17	1.21
IQ/DQ	65.19	1.80

For clarity, the following acronyms are used throughout the manuscript to refer to assessment instruments and outcome measures: *Autism Diagnostic Observation Schedule – Second Edition Social Affect* (ADOS-SA) score, *Autism Diagnostic Observation Schedule – Second Edition Total* (ADOS-TOT) score, *Child Behavior Checklist* Internalizing Problems (CBCL-INT) scale, *Child Behavior Checklist* Externalizing Problems (CBCL-EXT) scale, and *Child Behavior Checklist* Total Problems (CBCL-TOT) score. Cognitive functioning is typically assessed using an Intelligence Quotient (IQ). Sleep disturbances are assessed using the *Sleep Disturbance Scale for Children* Total score (SDSC-TOT), including Disorders of Initiating and Maintaining Sleep (DIMS), Disorders of Initiating Sleep (DIS), Disorders of Maintaining Sleep (DMS), Parasomnias (PARA), Sleep Breathing Disorders (SBD), Sleep–Wake Transition Disorders (SWTD), and Disorders of Excessive Somnolence (DOES). Parental stress is measured using the *Parenting Stress Index* Total score (PSI-TOT). Adaptive functioning is evaluated using the *Adaptive Behavior Assessment System*, Practical domain (ABAS-DAP) and the *Adaptive Behavior Assessment System*, Communication domain (ABAS-DAC).

The prevalence of sleep difficulties, defined as an SDSC total (SDSC-TOT) score >60, varied across age groups. In the 6–36 months group, 20% of participants showed clinically elevated SDSC total scores. In the 3–6-year-old group, clinically significant SDSC scores were observed in 16.5% of participants. The highest prevalence was found in the 6–18 years group, where 30% of children and adolescents exhibited SDSC total scores in the clinical range.

Pearson correlation analyses were conducted to examine the relationships between parenting stress and both parent-reported and clinician-administered child measures, to assess the degree of association among these constructs, and to evaluate potential shared-method variance when interpreting parent-reported outcomes. As shown in Table 4, parenting stress was primarily associated with parent-reported sleep disturbances and behavioral problems across age groups. In contrast, associations with clinician-administered measures of autism severity were weak or non-significant.

**Table 4:** Pearson correlations between parenting stress (PSI-total [PSI-TOT] score) and parent-reported and clinician-administered child measures across age groups.

Age group	Measure	Type of measure	*r* (PSI)	*p*-value
6–36 months	SDSC-TOT	Parent-report	0.12	0.37
	CBCL-TOT problems	Parent-report	0.32*	0.019
	ABAS-GAC	Parent-report	0.15	0.26
	ADOS2-TOT	Clinician-administered	−0.25	0.065
3–6 years	SDSC-TOT	Parent-report	0.37**	<0.001
	CBCL-TOT Problems	Parent-report	0.64**	<0.001
	ABAS-GAC	Parent-report	−0.42**	<0.001
	ADOS2-TOT	Clinician-administered	0.23*	0.030
≥6 years	SDSC-TOT	Parent-report	0.31*	0.012
	CBCL-TOT Problems	Parent-report	0.37**	0.005
	ABAS-GAC	Parent-report	−0.07	0.59
	ADOS2-TOT	Clinician-administered	0.08	0.54

*p < 0.05; **p < 0.01.

### 3.2 Multivariate analysis

#### 3.2.1 6–36 months

Multivariate analysis shows that for the model associated with the 6–36 months of age group ADOS-SA (*Λ* = 0.804 with *F* = 1,081; *p* < 0.005), ADOS-TOT (Λ = 0.741 with *F* = 1,544; *p* < 0.005), CBCL-INT (Λ = 0.715 with *F* = 1,762; *p* < 0.005), CBCL-EXT (Λ = 0.837 with *F* = 0,862; *p* < 0.005), CBCL-TOT (Λ = 0.716 with *F* = 1,754; *p* < 0.005) and QI (Λ = 0.703 with *F* = 1,868; *p* < 0.005), have statistically significant effect in the model.

Specifically, univariate analyses indicate that higher ADOS-TOT scores are associated with higher DIS (*β* = 2,404, *p* < 0.05) scores, whereas higher ADOS-SA scores are associated with lower DOES (*β* = −4,934, *p* < 0.05) scores. Higher CBCL-INT scores are associated with lower DMS (*β* = −1.641, *p* < 0.05), PARA (*β* = −0,812, p < 0.05), and SDSC-TOT (*β* = −1,144, p < 0.05) scores.

Higher CBCL-EXT scores are associated with lower DMS (*β* = −0,871, *p* < 0.05); CBCL-TOT scores are associated with higher DMS (*β* = 2,363, *p* < 0.05), PARA (*β* = 1,371, *p* < 0.05), and SDSC-TOT (*β* = 1,911, *p* < 0.05) scores.

Finally, higher IQ levels are associated with higher PARA (*β* = 0,253, *p* < 0.05) and DOES (*β* = 0,385, *p* < 0.05) scores (Tables 5, 6).

**Table 5:** Multivariate analysis 6–36 months.

/	DIS		DMS		SBD		PARA	
	*F*	*B* (SE)	*F*	*B* (SE)	*F*	*B* (SE)	*F*	*B* (SE)
Corrected model	1.806	/	2.226	/	1.389	/	3.032	/
Intercept	2.289	31.525 (20.838)	9.142	61.028 (20.184)	7.322	56.026 (20.706)	9.775	51.317 (16.413)
ADOS2-AS	2.982	–3.476 (2.013)	0.282	1.036 (1.949)	0.015	0.231 (2.000)	0.155	−0.624 (1.585)
ADOS2-RRB	0.000	–0.022 (1.549)	1.462	1.814 (1.500)	0.044	−0.323 (1.539)	0.565	0.917 (1.220)
ADOS2-TOT	5.650	2.404* (1.012)	0.170	–0.404 (0.980)	0.027	–0.165 (1.005)	0.715	–0.674 (0.797)
PSI-TOT	0.905	0.067 (.071)	0.595	–0.053 (0.069)	0.277	0.037 (0.070)	0.412	–0.036 (0.056)
CBCL-INT	1.959	–0.688 (0.491)	11.891	−1.641** (0.476)	3.056	−0.854 (0.488)	4.404	−0.812* (0.387)
CBCL-EXT	2.896	−0.726 (0.427)	4.437	−0.871* (0.413)	0.177	−0.179 (0.424)	2.482	−0.530 (0.336)
CBCL-TOT	3.415	1.296 (0.701)	12.111	2.363** (0.679)	2.086	1.006 (0.697)	6.166	1.371* (0.552)
ABAS-GAC	0.230	−0.126 (0.263)	0.277	−0.134 (0.255)	0.008	0.023 (0.262)	0.103	−0.067 (0.207)
ABAS-DAC	0.519	0.146 (0.202)	1.623	0.249 (0.196)	0.946	0.195 (0.201)	0.040	−0.032 (0.159)
ABAS-DAS	0.063	−0.056 (0.224)	0.346	−0.128 (0.217)	0.560	−0.167 (0.233)	0.018	0.024 (0.176)
ABAS-DAP	0.560	−0.45 (0.188)	0.311	−0.102 (0.182)	1.183	−0.203 (0.187)	0.170	−0.061 (0.148)
IQ/GDQ	1.518	0.153 (0.124)	0.027	-0.20 (0.120)	0.955	0.121 (0.124)	6.657	0.253* (0.098)
*R*^2^ (adjusted)	0.165		0.231		0.087		0.332	

*p < 0.05; **p < 0.01.

**Table 6:** Multivariate analysis 6–36 months.

/	DOES		SHY		TOT	
	*F*	*B* (SE)	*F*	*B* (SE)	*F*	*B* (SE)
Corrected model	2.129	/	0.502	/	2.331	/
Intercept	1.867	35.326 (25.854)	4.417	65.414 (31.125)	7.079	50.638 (19.032)
ADOS2-AS	3.905*	−4.934* (2.497)	0.031	0.526 (3.006)	1.272	–2.073 (1.838)
ADOS2-RRB	0.009	−0.187 (1.921)	0.561	–1.733 (2.313)	0.007	0.120 (1.414)
ADOS2-TOT	0.817	1.134 (1.255)	0.016	0.190 (1.511)	0.943	0.897 (0.924)
PSI-TOT	0.357	−0.052 (0.088)	0.046	−0.023 (0.106)	0.325	–0.037 (0.065)
CBCL-INT	0.101	0.194 (0.610)	0.729	−0.627 (0.734)	6.498	–1.144 * (0.449)
CBCL-EXT	0.807	−0.476 (0.530)	0.030	−0.110 (0.638)	3.212	−0.699 (0.390)
CBCL-TOT	0.811	0.783 (0.870)	0.565	0.787 (1.047)	8.906	1.911* (0.640)
ABAS-GAC	0.001	−0.011 (0.327)	0.108	0.129 (0.393)	0.112	−0.081 (0.240)
ABAS-DAC	0.224	−0.119 (0.251)	0.286	0.161 (0.302)	1.322	0.212 (0.185)
ABAS-DAS	0.683	−0.230 (0.278)	1.401	−0.396 (0.335)	2.038	0.212 (0.185)
ABAS-DAP	0.198	0.104 (0.233)	0.047	−0.061 (0.281)	0.063	−0.292 (0.205)
IQ/GDQ	6.226	0.385* (0.154)	0.194	0.082 (0.186)	3.182	−0.043 (0.172)
*R*^2^ (adjusted)	0.217		0.139		0.246	0.203 (0.114)

*p < 0.05.

#### 3.2.2 3–6 years

Multivariate analysis shows that for the model related to the 3–6-year-old group, only CBCL-TOT (Λ = 0.912 with *F* = 0,966; *p* < 0.05) has a statistically significant effect in the model. Specifically, univariate analyses indicate that higher CBCL-TOT scores are associated with higher SDSC-TOT scores (*β* = 0,634, *p* < 0.05) (Tables 7, 8).

**Table 7:** Multivariate analysis 3–6 years.

/	PARA		DIMS		SBD		DOES	
	*F*	*B* (SE)	*F*	*B* (SE)	*F*	*B* (SE)	*F*	*B* (SE)
Corrected model	2.422	/	2.702	/	0.580	/	0.789	/
Intercept	2.607	22.459 (13.909)	3.133	30.460 (17.208)	5.932	46.143 (18.945)	2.557	117.551 (73.515)
ADOS2-AS	0.719	0.889 (1.048)	1.171	0.536 (1.297)	0.038	−0.278 (1.428)	3.294	10.054 (5.540)
ADOS2-RRB	0.462	−0.636 (0.936)	0.004	−0.072 (1.159)	0.177	0.537 (1.276)	0.535	3.621 (4.950)
ADOS2-TOT	0.010	−0.045 (0.441)	0.416	−0.352 (0.545)	0.300	−0.329 (0.600)	2.981	-4.021 (2.329)
PSI-TOT	1.349	0.060 (0.052)	0.022	0.009 (0.064)	0.472	−0.049 (0.071)	0.186	−0.119 (0.275)
CBCL-INT	0.014	−0.15 (0.127)	0.019	–0.022 (0.157)	0.029	–0.030 (0.173)	0.086	–0.197 (0.671)
CBCL-EXT	0.090	–0.066 (0.220)	0.059	–0.066 (0.272)	0.032	0.053 (0.299)	0.234	0.562 (1.160)
CBCL-TOT	2.415	0.377 (0.242)	3.649	0.573 (0.300)	0.242	0.162 (0.330)	0.168	–0.524 (1.281)
ABAS-GAC	0.007	–0.024 (0.282)	0.001	0.012 (0.349)	0.287	0.206 (0.384)	0.843	1.369 (1.491)
ABAS-DAC	0.011	–0.015 (0.147)	0.184	–0.078 (0.182)	2.793	–0.335 (0.201)	0.082	–0.222 (0.779)
ABAS-DAS	0.329	–0.083 (0.145)	0.492	0.126 (0.180)	0.010	0.020 (0.198)	1.286	–0.871 (0.768)
ABAS-DAP	0.011	–0.015 (0.140)	0.132	–0.063 (0.173)	0.007	0.016 (0.190)	0.826	–0.672 (0.739)
IQ/GDQ	0.227	0.024 (0.050)	0.064	–0.016 (0.062)	0.793	0.061 (0.068)	3.359	–0.485 (0.265)
*R*^2^ (adjusted)	0.162		0.188		–0.061		–0.030	

**Table 8:** Multivariate analysis 3–6 years.

/	SHY		NRS		TOT	
	*F*	*B* (SE)	*F*	*B* (SE)	*F*	*B* (SE)
Corrected model	1.690	/	2.046	/	3.265	/
Intercept	3.525	31.479 (16.767)	10.177	50.120 (15.711)	3.394	29.167 (15.832)
ADOS2-AS	0.029	–0.214 (1.263)	0.966	1.163 (1.184)	0.414	0.767 (1.193)
ADOS2-RRB	1.413	1.342 (1.129)	3.309	−1.924 (1.058)	0.775	−0.938 (1.066)
ADOS2-TOT	0.694	−0.442 (0.531)	2.290	−0.268 (0.498)	0.172	−0.208 (0.502)
PSI-TOT	0.598	0.049 (0.063)	0.570	0.044 (0.059)	0.036	0.011 (0.059)
CBCL-INT	0.023	0.023 (0.153)	3.281	−0.260 (0.143)	0.598	−0.112 (0.145)
CBCL-EXT	0.160	−0.106 (0.265)	0.092	−0.075 (0.248)	0.133	−0.091 (0.250)
CBCL-TOT	1.268	0.329 (0.292)	2.568	0.439 (0.274)	5.279	0.634* (0.276)
ABAS-GAC	0.629	−0.270 (0.340)	0.404	−0.203 (0.319)	0.168	−0.131 (0.321)
ABAS-DAC	0.328	0.102 (0.178)	0.071	0.044 (0.166)	0.157	−0.066 (0.168)
ABAS-DAS	0.869	0.163 (0.175)	0.192	0.072 (0.164)	1.205	0.182 (0.165)
ABAS-DAP	0.000	−0.003 (0.169)	0.074	0.043 (0.158)	0.002	0.007 (0.159)
IQ/GDQ	0.426	−0.039 (0.060)	0.502	0.040 (0.057)	0.124	0.020 (0.057)
*R*^2^ (adjusted)	0.086		0.125		0.236	

*p < 0.05.

#### 3.2.3 6–18 years

Multivariate analysis shows that for the model associated with the 6–15 years age group ADOS-SA (Λ = 0.813 with *F* = 1,446; *p* < 0.005), PSI-TOT (Λ = 0.613 with *F* = 3,970; *p* < 0.005), ABAS-DAP (Λ = 0.851 with *F* = 1,097; *p* < 0.005), ABAS-DAC (Λ = 0.852 with *F* = 1,092; *p* < 0.005), have statistically significant effect in the model.

Specifically, univariate analyses indicate that higher ADOS-SA scores are associated with lower SWTD scores (*β* = −4,737, *p* < 0.05). Higher PSI-TOT scores are related to higher DIMS (*β* = 0,254, *p* < 0.05), higher SBD (*β* = 0,106, *p* < 0.05), and higher SWTD (*β* = 0,180, *p* < 0.05) scores.

Moreover, higher ABAS-DAP scores also have a significant effect on SBD (*β* = 0,532, *p* < 0.05) scores and DOES (*β* = 0,350, *p* < 0.05) scores (Tables 9, 10).

**Table 9:** Multivariate analysis 6 + years.

/	DIMS		SBD		DA		SWTD	
	*F*	*B* (SE)	*F*	*B* (SE)	*F*	*B* (SE)	*F*	*B* (SE)
Corrected model	2.243	/	1.426	/	1.496	/	1.540	/
Intercept	0.336	17.717 (30.580)	1.865	29.551 (21.641)	0.199	−12.742 (28.529)	4.202	53.266 (25.966)
ADOS2 AS	0.001	−0.058 (2.161)	1.557	−1.908 (1.529)	0.240	−0.988 (2.016)	6.664	−4.737* (1.835)
ADOS2-RRB	0.316	0.771 (1.370)	2.339	−1.483 (0.970)	0.085	0.0.372 (1.278)	1.122	−1.232 (1.163)
ADOS2-TOT	0.191	−0.395 (0.903)	1.593	0.806 (0.639)	0.174	0.351 (0.842)	1.902	1.057 (0.767)
PSI-TOT	10.751	0.254* (0.078)	3.732	0.106* (0.055)	2.724	0.119 (0.072)	7.510	0.180* (0.066)
CBCL-INT	0.618	0.219 (0.279)	1.097	0.207 (0.197)	0.064	−0.066 (0.260)	0.007	−0.020 (0.237)
CBCL-EXT	2.148	−0.724 (0.494)	0.173	0.146 (0.350)	0.124	−0.162 (0.461)	0.021	0.060 (0.414)
CBCL-TOT	1.662	0.655 (0.508)	0.354	−0.214 (0.360)	0.860	0.440 (0.474)	0.000	0.006 (0.431)
ABAS-GAC	2.970	−1.050 (0.609)	1.900	−0.594 (0.431)	1.173	−0.615 (0.568)	0.170	−0.213 (0.517)
ABAS-DAC	0.693	0.232 (0.278)	1.402	0.233 (0.197)	1.528	0.321 (0.260)	0.449	0.158 (0.236)
ABAS-DAS	1.408	0.324 (0.273)	0.048	−0.042 (0.193)	3.389	0.468 (0.254)	0.199	0.103 (0.232)
ABAS-DAP	1.303	0.399 (0.350)	4.625	0.532* (0.248)	0.775	0.287 (0.326)	0.021	0.043 (0.297)
IQ/GDQ	0.830	0.123 (0.135)	0.150	0.037 (0.096)	1.709	0.165 (0.126)	0.234	−0.056 (0.115)
*R*^2^ (adjusted)	0.194		0.076		0.088		0.095	

*p < 0.05.

**Table 10:** Multivariate analysis 6 + years.

/	DOES		IPN		TOT	
	*F*	*B* (SE)	*F*	*B* (SE)	*F*	*B* (SE)
Corrected model	2.417	/	1.133	/	1.957	/
Intercept	2.710	31.124 (18.905)	1.167	24.154 (22.363)	0.150	9.560 (24.651)
ADOS2 AS	2.251	−2.004 (1.336)	1.666	−2.040 (1.580)	0.900	−1.653 (1.742)
ADOS2-RRB	0.514	0.608 (0.847)	0.300	−0.548 (1.002)	0.132	0.401 (1.105)
ADOS2-TOT	0.000	−0.010 (0.558)	1.466	0.799 (0.660)	0.041	0.148 (0.728)
PSI-TOT	0.091	0.014 (0.048)	0.002	0.002 (0.057)	2.809	0.105 (0.063)
CBCL-INT	1.914	0.239 (0.173)	1.369	0.239 (0.204)	1.511	0.276 (0.225)
CBCL-EXT	0.720	−0.259 (0.305)	0.817	−0.326 (0.361)	1.131	−0.423 (0.398)
CBCL-TOT	0.688	0.260 (0.314)	1.115	0.392 (0.372)	1.416	0.487 (0.410)
ABAS-GAC	0.643	−0.302 (0.377)	1.003	−0.446 (0.445)	2.865	−0.831 (0.491)
ABAS-DAC	4.140	0.350 (0.172)	2.869	0.345 (0.203)	3.370	0.412 (0.224)
ABAS-DAS	0.251	−0.084 (0.169)	0.089	0.060 (0.199)	0.751	0.191 (0.220)
ABAS-DAP	0.277	0.114* (0.216)	1.561	0.320 (0.256)	1.463	0.341 (0.282)
IQ/GDQ	0.358	0.050 (0.084)	0.859	−0.092 (0.099)	0.938	0.106 (0.109)
*R*^2^ (adjusted)	0.215		0.025		0.156	

*p < 0.05.

## 4 Discussion

The current study assessed the prevalence of sleep problems in a relatively large group of autistic children and adolescents. The aim of the present study was to explore the age-specific relationship between sleep disorders, cognitive development, severity of ASD, behavioral and emotional symptoms, and Parenting stress in a sample of autistic children.

To achieve the study’s aim, the sample was divided into three subgroups based on different age groups identified by the SDSC tool used. The effects of clinical features on various sleep domains across different age groups were evaluated and discussed.

### 4.1 Sleep and autism: developmental insights from children aged 6–36 months

We examined the relationship between sleep disorders and clinical features such as ASD symptomatology, behavioral challenges, and cognitive abilities. We found that children with more pronounced autism-related behaviors often struggled with initiating sleep. Behavioral problems were linked to distinct sleep patterns, with emotional regulation difficulties playing a key role. Additionally, higher cognitive abilities were associated with specific sleep disorders, suggesting a complex relationship between intelligence and sleep architecture. These findings highlight the relationship between developmental and behavioral factors in shaping sleep patterns during early childhood.

We found a positive association between higher ADOS-TOT scores and higher Disorders of Initiating Sleep scores in children aged 6–36 months. This finding highlights a connection between higher autism symptomatology and increased difficulties in initiating sleep in early childhood.

Our findings are consistent with existing literature, which identifies difficulties with initiating sleep as a prevalent and core characteristic of ASD (Sommers et al., 2024; Richdale, 1999; Liu et al., 2006; Krakowiak et al., 2008; Díaz-Román et al., 2018). However, the reasons for their co-occurrence remain poorly understood. One possible explanation is that they may arise from shared underlying etiological factors (Taylor et al., 2022).

Another possible explanation might concern the main features of ASD. ADOS TOTAL score is used to classify the condition into autism, autism spectrum, or non-autism (Gotham et al., 2009; Park et al., 2018). Higher ADOS-TOT scores reflect more pronounced autism-related behaviors. Autistic children often show heightened sensitivity to sensory stimuli (Sapey-Triomphe et al., 2023), such as light, noise, or bedding textures, which can interfere with their ability to relax and fall asleep. Behavioral and cognitive rigidity represent other key symptoms in autistic children (Lage et al., 2024). Cognitive flexibility refers to the ability to switch between different tasks or strategies and adapt to changing situations (Lage et al., 2024; Dajani and Uddin, 2015). For autistic people, predictability plays a pivotal role. Consistent routines produce a structured framework that helps them anticipate what comes next. According to this, any disruptions to everyday routines, even in terms of bedtime rituals and sleep environment, may increase stress and interfere with the phase of falling asleep.

In addition, delays in language development in autistic children are commonly observed (Singhi and Malhi, 2023). As a result, sleep difficulties might show when language impairment and communication challenges possibly reflect difficulties in expressing discomfort, fear, or other needs. This finding is consistent with previous polysomnographic studies showing that variations in slow-wave (NREM) and REM sleep are associated with core ASD symptoms and behavioral profiles, suggesting that atypical sleep stage composition may relate to characteristic neurobehavioral patterns in ASD (Kawai et al., 2023). However, our study relied on the *Sleep Disturbance Scale for Children* (SDSC), a validated questionnaire assessing subjective and behavioral aspects of sleep. Consequently, while our results align with polysomnographic findings, they cannot be interpreted as direct evidence of neurobiological alterations; rather, reflect parent-reported sleep difficulties relative to age-normed expectations.

Moreover, we found an association between higher ADOS-SA scores and lower Disorders of Excessive Somnolence scores in children aged 6–36 months. This finding reflects a behavioral pattern in which greater social-affective difficulties—such as reduced social engagement—were associated with fewer signs of excessive somnolence during the day.

As a matter of fact, children with higher ADOS-SA scores show reduced interest in their social environment (Gotham et al., 2007; Hus et al., 2014), including socioemotional interactions and play-oriented activities. Epidemiological research has shown that both social and physical environmental factors can significantly influence sleep patterns and contribute to sleep disorders (Johnson et al., 2018). Limited social interaction may be associated with lower emotional and cognitive arousal, making it easier to transition into sleep and reducing nighttime disturbances.

We also found that higher CBCL-INT scores were associated with lower scores on Disorders of Maintaining Sleep, Parasomnias, and Total Sleep Disorders in children aged 6–36 months. From a behavioral perspective, this finding suggests that higher levels of internalizing symptoms were associated with fewer difficulties in maintaining sleep, fewer parasomnia-related behaviors, and fewer overall sleep disruptions in early childhood. These findings diverge from existing literature (Cohen et al., 2014; Gisbert Gustemps et al., 2021; Favole et al., 2023) and require careful interpretation. It should be noted that the internalizing subscale of the CBCL version for children aged 1.5–5 years includes a small number of items assessing sleep-related problems, which may partially overlap with SDSC measures. However, these items refer to specific behavioral manifestations of sleep difficulties and represent only a limited component of the broader internalizing domain. In contrast, the SDSC provides a more detailed and domain-specific assessment of sleep disturbances. Several hypotheses may explain this finding.

First, the discrepancy may arise from differences in caregivers’ perceptions and interpretations. Internalizing problems are often less visible than externalizing behaviors like aggression or hyperactivity (Aguilar-Yamuza et al., 2023). Moreover, internalizing symptoms in this age group may overlap with core ASD symptoms. Autistic children aged 6–36 months often exhibit social withdrawal (Zhou and Buss, 2021) that caregivers may perceive as internalizing behavior rather than recognizing it as potential signs of ASD. These emerging difficulties could lead parents to focus on behavioral challenges rather than recognizing sleep-related issues. Furthermore, language impairments commonly observed in autistic children (Singhi and Malhi, 2023) may hinder caregivers from accurately identifying parasomnias or other sleep disorders.

Second, the relationship between internalizing symptoms and sleep difficulties may be mediated by factors not directly evaluated in our study, such as temperament and parenting practices. Temperament traits are closely linked to behavioral and emotional regulation, playing a mediating role (Sacrey et al., 2022). Internalizing symptoms in childhood are linked to a higher level of effortful control, leading to a behavioral pattern of overcontrol (Tandon et al., 2009) that might be extended to sleep. Parasomnias often occur during NREM sleep, particularly in its deeper stages (Howell, 2012). Consequently, conditions that lead to frequent nighttime awakenings (such as higher levels of control) may reduce the time spent in these sleep stages. This idea aligns with the findings of Wu et al. (2022), demonstrating an inverse relationship between sleep duration and internalizing symptoms.

Parenting behaviors may also influence the relationship between internalizing symptoms and sleep (van der Sluis et al., 2015). Parents’ perception of their children’s quality of sleep is based primarily on whether they wake up during the night (Gomes and Martins, 2021). Specific parenting strategies may be associated with underreporting of children’s sleep difficulties, leading caregivers to minimize their children’s sleep difficulties.

Furthermore, some items within the CBCL, particularly for internalizing behaviors, already capture elements related to sleep (Gregory et al., 2011) which could also overlap with manifestations of sleep disturbance. As a result, these symptoms may contribute to CBCL-INT scores without being recognized as distinct sleep disorders associated with SDSC scores. Sleep problems could be underreported or viewed as part of the child’s overall emotional struggles rather than distinct sleep disorders.

These findings highlight the need for further research to explore the intricate interactions between internalizing symptoms, temperament, parenting practices, and sleep in young autistic children.

When examining externalizing behaviors, our study revealed an association between higher CBCL-EXT scores and lower Disorders of Maintaining Sleep scores in children aged 6–36 months. From a behavioral perspective, greater expression of externalizing symptoms (such as aggression and hyperactivity) was associated with fewer observed problems related to maintaining sleep. One possible explanation regards the strength of externalizing symptoms. Externalizing behaviors, such as hyperactivity, require significant energy throughout the day. This may result in physical overtiredness, which can make these children fall asleep more easily and experience better sleep maintenance due to fatigue (Weiss and Salpekar, 2010). Another potential explanation regards parenting point of view. Several studies show how sensitive parenting during the first 3 years of a child’s life is associated with fewer externalizing behaviors in early and middle childhood (Cloud et al., 2024; Bradley and Corwyn, 2008; Wang et al., 2013; Shaw et al., 1994). Parental sensitivity refers to the ability to recognize and respond appropriately to their child’s needs (Wang et al., 2013). Caregivers, who frequently deal with externalizing behaviors, may focus on daytime challenges, thereby unintentionally neglecting sleep-related concerns, resulting in a less accurate assessment of the child’s sleep issues. As a result, they may perceive the child’s sleep as less problematic, particularly compared with the behavioral challenges during the day.

Our analysis revealed a positive association between CBCL-TOT scores and Total Sleep Disorders scores in children aged 6–36 months. The CBCL-TOT scores reflect not only behavioral and emotional difficulties but also children’s challenges with emotional regulation (Adynski et al., 2024), critical for sleep (Lollies et al., 2022). Difficulties in emotional regulation are closely linked to difficulties in maintaining restorative sleep patterns. Moreover, this relationship may be bidirectional: poor sleep exacerbates emotional and behavioral issues, while emotional and behavioral difficulties in turn disrupt sleep, building a reinforcing cycle (Kahn et al., 2013).

Finally, we found an association between higher levels of IQ and higher levels of Parasomnias and Disorders of Excessive Somnolence scores in children 6–36 months. Thieux et al. (2024) reported that children with high IQ exhibit increased rapid eye movement (REM) sleep, particularly during the later part of the night, suggesting a potential relationship between cognitive functions and sleep architecture. However, the sleep questionnaire utilized in our study was not designed to differentiate between specific parasomnia subtypes, including those associated with REM and NREM sleep phases (Bruni et al., 1996; Romeo et al., 2021; Romeo et al., 2013). While existing literature has identified associations between specific parasomnia typologies and sleep phases (Markov et al., 2006), the relationship between intellectual functioning, parasomnias, and excessive somnolence remains unclear. This highlights the need for further research to elucidate the relationship between cognitive abilities, parasomnia characteristics, and excessive somnolence.

### 4.2 Sleep and autism: developmental insights from children aged 3–6 years

Our analysis uncovered a positive association between CBCL-TOT and total Sleep Disorders scores in children aged 3–6 years, consistent with our previous findings in children aged 6–36 months. This connection supports the regulation hypothesis and suggests the presence of stable trajectories linking behavioral and sleep difficulties across early developmental stages (Lollies et al., 2022).

In this regard, our results align with several studies (Taylor et al., 2012; Sikora et al., 2012; Berkovits et al., 2017; Zaidman-Zait et al., 2020). Emotional regulation and sleep difficulties are common in young children with neurodevelopmental disorders. However, the direction of this relationship remains under investigation (Favole et al., 2023). Emotion regulation is a complex process involving the monitoring, evaluation, and modulation of emotional responses (Gross, 1998), including reducing emotional arousal (Gratz and Roemer, 2008). When individuals struggle to regulate their emotional or physiological states, difficulties in falling or maintaining sleep may be observed. As a matter of fact, the presence of emotional dysregulation has been demonstrated in patients with sleep disorders (Palagini et al., 2017). In particular, sleep disorders were closely linked to emotional dysregulation in young autistic children, both in cross-sectional and longitudinal analyses. Moreover, emotional dysregulation was associated with behavioral challenges, including sleep difficulties and internalizing symptoms.

Moreover, several studies suggest that the core features of ASD may contribute to the development of emotional dysregulation, with repetitive behaviors, social difficulties, and alexithymia acting as key promoting factors (Dell’Osso et al., 2023; Gormley et al., 2022; Morie et al., 2019; Samson et al., 2014), possibly explaining the frequent co-occurrence of ASD and sleep disorders.

### 4.3 Sleep and autism: developmental insights from children aged 6–18 years

We identified significant associations between sleep disorders and developmental, behavioral, and parental factors.

The analysis identified an association between higher ADOS-SA scores and lower Sleep–Wake Transition Disorders scores in children aged 6–18 years. These results could be explained by the evidence that autistic children with high levels of autistic symptomatology, including impairments in social interaction and communication, may prefer rigid routines and predictable environments (Gotham et al., 2007), which are factors that promote better sleep quality and a smoother transition between sleep and wakefulness (Mindell et al., 2015). The predictability and structure of routines could reduce anxiety (Lang et al., 2022; Sellick et al., 2021), which may otherwise impede the transition between sleep and wakefulness.

Furthermore, we found an association between higher PSI-TOT scores and higher Disorders of Initiating and Maintaining Sleep, Sleep Breathing Disorders, and Sleep Waking Transition Disorders scores in children aged 6–18 years. Children with social difficulties and behavioral problems can be associated with higher-level parenting levels of parenting stress (Neece et al., 2012; Ribas et al., 2024). Accordingly, caregivers may struggle to provide emotional support and to employ effective strategies for regulating their child’s behavior and emotions. When parents are overwhelmed, they may have fewer resources—both physically and emotionally—to implement high-quality regulatory strategies. This lack of regulation and support may be associated with greater difficulties in managing sleep-related issues (Goldman et al., 2012; Iwamoto et al., 2023).

Finally, our analysis showed a positive association between higher ABAS-DAP scores and Sleep Breathing Disorders and Disorders of Excessive Somnolence scores, suggesting a potential link between adaptive behavior and sleep regulation. The Practice Domain of the ABAS-II focuses on the everyday skills required for living, including personal care and task management across different settings.

Specific sleep disorders may impact adaptive behavior (Sikora et al., 2012; Hammond et al., 2022). However, the nature of this relationship remains unclear, with limited evidence on whether sleep disorders may contribute to difficulties in adaptive behavior or if impairments in adaptive behavior increase the likelihood of sleep disorders. Our proposed explanation suggests an indirect association, where adaptive behavior influences sleep patterns, which may be influenced by other factors. Notably, higher levels of adaptive behavior are often associated with higher IQ (Tassé and Kim, 2023). Higher IQ in autistic children is frequently linked to comorbidities such as anxiety or mood disorders (Mingins et al., 2021) which may be associated with variations in sleep patterns. Adolescents and school-age children have the highest prevalence of clinical and subclinical anxiety compared to other autistic age groups (Zaboski and Storch, 2018). Anxiety disorders are characterized by a wide range of symptoms, including excessive worry, social and performance fears, unexpected or triggered panic attacks, anticipatory anxiety, and avoidance behaviors. These conditions may also manifest as hyperarousal and difficulty concentrating (Szuhany and Simon, 2022; Robinson et al., 2013) possibly leading to higher sleep disorders.

Notably, no significant associations were found between CBCL scores and sleep disorders in this age group, highlighting the complex and multifaceted nature of sleep-related challenges in autistic children and behavioral issues. This suggests that other factors, beyond general behavioral and emotional difficulties, may play a more prominent role in shaping sleep patterns at later developmental stages.

## 5 Conclusion

This study highlights the association between clinical features and sleep disorders across different age groups in autistic children. The analysis identified age-specific differences in the relationships between ASD symptoms, emotional and behavioral challenges, and sleep difficulties.

Our findings suggest that sleep disorders in autistic children are not exclusively influenced by the core features of the disorder. A dynamic interplay of developmental and environmental factors over time was observed. The study emphasizes the importance of considering age-specific factors when examining sleep patterns in autistic children. The impact of behavioral and emotional challenges on sleep may vary across different developmental stages. Due to this variability, the study underscores the need for age-specific analysis in line with children’s changing developmental trajectories and symptom profiles.

Furthermore, the impact of parenting stress on sleep difficulties highlights the importance of supporting families to improve sleep outcomes in autistic children.

Given the heterogeneity of sleep disorders across age groups, tailored interventions are essential for optimizing sleep quality and enhancing the quality of life for both children and their caregivers. A multidisciplinary approach that integrates behavioral, medical, and environmental strategies can significantly enhance sleep quality, thereby improving daytime functioning and overall quality of life for both children and their families. Future research should continue to explore individualized intervention models that integrate behavioral, pharmacological, and technological solutions to optimize sleep outcomes in this population.

### 5.1 Study limitations and future directions

The current study has several limitations. First, the study’s cross-sectional and retrospective design precludes causal inferences regarding the association between sleep disorders and clinical features. Longitudinal studies are essential to clarify the directionality of these relationships.

Another limitation concerns the use of parent-report measures and the lack of objective sleep measures, which may introduce reporting bias and limit the accuracy of assessing children’s sleep patterns, as suggested by small-to-moderate associations between parenting stress and parent-reported outcomes. However, autism severity and cognitive functioning were assessed by clinicians, limiting the impact of shared-method variance for these core clinical variables.

Sleep was assessed exclusively via parent-report questionnaires. While objective measures such as actigraphy or polysomnography were not included, the *Sleep Disturbance Scale for Children* (SDSC) has been validated in the Italian population, including children with ASD, supporting its reliability and clinical relevance (Mignolli et al., 2022). Studies comparing SDSC scores with objective sleep measures, including actigraphy and polysomnography, indicate moderate concordance, demonstrating that parent-reported difficulties generally reflect actual sleep problems, though the correlation is not perfect (Pinghini et al., 2025). This highlights that SDSC and objective measures capture complementary aspects of sleep: SDSC reflects perceived behavioral and clinical difficulties, whereas actigraphy and polysomnography measure physiological sleep parameters. Incorporating objective assessments in future research would strengthen the evaluation of sleep patterns and reduce potential reporting bias.

Furthermore, the potential impact of biological, environmental, and familial factors, including temperament, daily routines, and socioeconomic status, was not assessed. Parental bedtime routines and sleep-related parenting practices were not investigated. Previous studies in at-risk populations have shown that parental sleep routines can significantly influence children’s sleep patterns and may also be associated with language development (Zuccarini et al., 2024; Sansavini et al., 2022).

Additionally, common comorbidities in ASD, such as anxiety, depression, and epilepsy, were not specifically addressed. Future research should include these variables.

The role of parenting stress warrants further investigation. Parenting stress interacts bidirectionally with child sleep disorders and behavioral challenges, creating a complex feedback loop. Future research should focus on clarifying these dynamics, particularly by examining the mediating or moderating role of caregiver stress in the relationship between child behavior and sleep disorders.

Although non-verbal IQ (Leiter-3), full-scale IQ (WISC-IV), and developmental quotient (Griffiths) measure overlapping cognitive constructs, research has shown that even theoretically similar constructs derived from different cognitive batteries may not be fully equivalent due to differences in test composition, normative samples, and relative weighting of cognitive domains (Salthouse, 2014).

Finally, the lack of significant associations between CBCL scores and sleep disorders in older children suggests that other factors may shape sleep patterns during adolescence, warranting further investigation.

Despite these limitations, the study has notable strengths. First, the sample was homogeneous across different age groups. The inclusion of both standardized questionnaires and parent-report measures strengthened the reliability of the findings, reflecting both clinical observations and real-world experiences.

Additionally, the study examined multiple domains of development, improving the accuracy of the findings and reducing the impact of confounding variables.

## Data Availability

The raw data supporting the conclusions of this article will be made available by the authors, without undue reservation.

## Glossary

ADOS-SA: Autism Diagnostic Observation Schedule – Second Edition, Social Affect score
ADOS-RRB: Autism Diagnostic Observation Schedule – Second Edition, Restricted and Repetitive Behaviors score
ADOS-TOT: Autism Diagnostic Observation Schedule–Second Edition, Total score
CBCL-INT: Child Behavior Checklist, Internalizing Problems scale
CBCL-EXT: Child Behavior Checklist, Externalizing Problems scale
CBCL-TOT: Child Behavior Checklist, Total Problems score
IQ: Intelligence Quotient
DQ/GDQ: General Developmental Quotient
SDSC-TOT: Sleep Disturbance Scale for Children, Total score
DIMS: Disorders of Initiating and Maintaining Sleep
DIS: Disorders of Initiating Sleep
DMS: Disorders of Maintaining Sleep
PARA: Parasomnias
SBD: Sleep Breathing Disorders
SWTD: Sleep–Wake Transition Disorders
DOES: Disorders of Excessive Somnolence
NRS: Non-Restorative Sleep
PSI-TOT: Parenting Stress Index, Total score
ABAS-GAC: Adaptive Behavior Assessment System, General Adaptive Composite
ABAS-DAC: Adaptive Behavior Assessment System, Communication domain
ABAS-DAS: Adaptive Behavior Assessment System, Social domain
ABAS-DAP: Adaptive Behavior Assessment System, Practical domain
F: F statistic
B: Unstandardized regression coefficient
SE: Standard Error
R^2^: Coefficient of determination
r: Pearson’s correlation coefficient
p: *p*-value
